# EQ-5D-5L reference values for the German general elderly population

**DOI:** 10.1186/s12955-021-01719-7

**Published:** 2021-03-06

**Authors:** Ole Marten, Wolfgang Greiner

**Affiliations:** School of Public Health, Bielefeld University, Universitaetsstrasse 25, 33615 Bielefeld, Germany

**Keywords:** Population norms, Reference values, EQ-5D-5L, Health-related quality of life, Health state utilities

## Abstract

**Background:**

Reference values are a helpful tool to facilitate comparisons of sampled values against a specified reference population. The aim is to describe the health profile and to provide visual analogue scale (EQ VAS) and utility reference values for the EQ-5D-5L from a normative sample of the general elderly population (65+) in Germany.

**Methods:**

We analysed a sub-set of data from the German EQ-5D-5L valuation study using self-reported information based on EQ-5D-5L. We examined the share of respondents in each severity level per dimension as well as means, standard deviations (SD) and 95% confidence intervals for the index and EQ VAS values stratified by age groups and gender. Age was categorised in four groups (65–69, 70–74, 75–79 and > 79 years) to facilitate a more detailed examination of age-related health-related quality of life (HRQoL).

**Results:**

The average index and EQ VAS scores were 0.84 (SD 0.22) and 73.2 (SD 18.5), respectively. In total, 21.4% reported no problems in all dimensions. With higher age, health problems were reported more frequently, which, in turn, lead to monotonically decreasing index and EQ VAS values. Overall, men reported fewer problems than women and this difference was largest beyond the age of 80.

**Conclusion:**

HRQoL in the oldest old appears to be less stable and differs from the young elderly. However, the conventional age categorisation of earlier population norms studies seems to mask these differences. Hence, the more detailed provision of EQ-5D-5L reference values for the elderly population seems helpful for future German studies.

## Background

The measurement and valuation of health-related quality of life (HRQoL) have become two of the most important components for the assessment of health and social care as well as public health interventions [1, 2], however, with limited applicability within the latter two sectors, since benefits of interventions may also be non-health-related [3, 4]. Nonetheless, using measures of HRQoL such as the EQ-5D to describe the benefits of treatments in all three sectors is still common and even recommended by the National Institute for Health and Care Excellence [5, 6]. The EQ-5D is a well-established and extensively used measure of HRQoL developed by the EuroQol Group [7, 8]. It can be characterised as a succinct and generic instrument, intended to measure health and deviations from it [9]. The EQ-5D has evolved to become one of the most widely used instrument to operationalise utilities for use in economic evaluation [10, 11]. However, informing resource allocation decisions is not the sole use of the instrument. It is further used for clinical appraisal, in epidemiological studies, in population health surveys and as a routine outcome measure in health care [9, 12–14]. The EQ-5D consists of a classification system with five dimensions (mobility, self-care, usual activities, pain or discomfort and anxiety or depression) and a subjectively rated visual analogue scale (EQ VAS) [15, 16]. The instrument provides a variety of information in terms of a descriptive health profile, the individual EQ VAS rating and an index score [17].

Health information based on these three EQ-5D components can be analysed and interpreted on an individual level. However, it can also be summarised and described at a population level. Such a set of aggregate comparator data is referred to as reference values or population norms and is useful for clinicians and health economists to compare sampled values against a specified reference group, e.g. the general population, to determine deviations in health [17]. Similarly, reference values may be used to populate health economic models with health state values [2]. Reference values generally refer to a defined population. Primarily, definition is based on regional aspects, where reference values are usually provided for the general population of that area, e.g. for the USA, Germany, Indonesia or South Australia [18–21]. Similarly, the reference population may be further stratified to represent diseases, i.e. patient groups, or socio-demographic groups [22, 23]. Furthermore, norms data may be derived based on different instruments such as the EQ-5D-3L and 5L, since those differ with regard to the provided information [24–26].

Several studies found that higher age is significantly associated with lower HRQoL as measured by the EQ-5D-5L [14, 19, 27]. Moreover, there is further evidence to suggest that variation in HRQoL is large in the elderly, which is even more pronounced in the oldest old [28, 29]. With regard to Germany, two sets of preference-based reference values were identified. Grochtdreis et al. [19] published EQ-5D-5L norms data for the German general population including elderly respondents, but using only two broad age bands for those aged 65 years and above. Secondly, König et al. [23] published reference values for the oldest-old (85+) in Germany based on EQ-5D-3L, which are, however, practically restricted by the underlying value set used to derive utilities. Given the expected increase in the proportion of elderly in Germany [30], we would like to argue that additional reference values based on EQ-5D-5L for elderly men and women of narrower age groups may be helpful to interpret changes in reported health more adequately, in particular, if returning to perfect health does not seem feasible [31]. Hence, the aim of this study is to provide reference values for Germany for the general elderly population, which are based on EQ-5D-5L using smaller age bands as compared to conventional population norms.

## Methods

### Data

The underlying data for this study originates from the German EQ-5D-5L valuation study conducted by Ludwig et al. [32], which had the primary aim to elicit preferences over EQ-5D-5L health states from a minimum sample of 1000 respondents from the German general population. The data was collected between December 2014 and March 2015 by a market research company (Kantar Health) using computer-assisted personal interviews. A quota-based sampling approach was used to obtain a representative sample with respect to age, gender, educational attainment and employment status for the German population. The respective quotas were based on German official statistics [33]. In order to ensure a geographical spread, sample recruitment and interviews were conducted in the cities of Berlin, Leipzig, Hamburg, Bielefeld, Munich and Frankfurt as well as in their surrounding areas. For the aim of this study we exploit the fact that as part of the original study respondents were asked to self-rate their health based on EQ-5D-5L. Moreover, respondents answered detailed background questions. The total sample comprised 1,158 interviews across all age bands [32]. In order to generate reference values for the German general elderly population we use a subset of the data only including respondents being 65 years and above; no further restrictions were made with respect to the sampling criteria in order to maintain the sample’s representativeness.

### Variables

Respondents answered the EQ-5D-5L to self-assess their health status along with detailed socio-demographic background questions [32]. The EQ-5D-5L has two components. First, the health state classification system consists of five dimensions: mobility (MO), self-care (SC), usual activities (UA), pain or discomfort (PD) and anxiety or depression (AD), where each can be described by five severity levels ranging from 1-‘no problems’ to 5-‘unable to/extreme problems’; thus, distinguishing 3,125 unique health states. Secondly, each respondent subjectively rated his overall health on the EQ VAS ranging from 0 to 100 labelled as ‘the worst health you can imagine’ and ‘the best health you can imagine’, respectively [8].

Further, the EQ-5D-5L health states can be summarised by a single index value on an interval scale being anchored at 1 for full health and 0 for being dead. EQ-5D index values can be derived using preference weights from the general population, which reflect the severity of the corresponding health state. We used the recommended value set for Germany by Ludwig et al. [32] to calculate the index value for each respondent. Generally, the German EQ-5D-5L value set covers values from 1 to −0.661, which is the worst possible health state with all dimensions being answered with level 5 [32]. The generated utilities are a third major information component used to summarise health of the sample [15].

Moreover, the survey included rich sociodemographic information on the respondents’ age, gender, occupational status, education level, income, marital status, religion and migration background. Available background information further relates to the area of residence, overall life satisfaction as well as financial security (both rated on a 0–10 Likert scale), whether the respondent is caring for someone or has experience with severe illness himself or within his family. Due to the interviewer-based approach information on all variables is complete for the entire sample [32].

### Analysis

We analysed the self-report health profile data by examining the proportion of respondents at each level of the EQ-5D dimensions for (i) the total sample and (ii) stratified by age (65–69, 70–74, 75–79 and > 79 years) and gender. We further calculated the percentage of respondents reporting any problem in the five dimensions and inspected commonly reported health states. For the reference values, we calculated EQ-5D-5L index values and present EQ VAS values for the total sample and stratified by age groups and gender using descriptive statistics [34]. Results are presented as means with standard deviations (SD) and the 95% confidence interval (CI) around the mean.

Since the EQ-5D-5L utilities and EQ VAS values were non-normally distributed (Shapiro–Wilk test), we used the non-parametric Kruskal–Wallis and Mann–Whitney-U tests to test for differences in the EQ VAS and EQ-5D-5L index value between age groups and gender, respectively. For statistical testing in two categorical variables we used Chi square tests to compare between groups. Differences between groups were considered statistically significant at a significance level of α = 0.05. All statistical analysis was carried out using STATA 16 [35].

## Results

### Sample characteristics

The normative sample was representative for the German elderly population with respect to age groups, sex, marital status, education and area of residence (Table 1). In total N = 290 respondents were 65 years and above and, hence, met the inclusion criteria for this reference value study. Respondents were aged 65–93 years with a mean age of 73.1 years (SD 5.7). The sample included slightly more women (54.5%). The regional distribution as well as the area of residence for men and women was similar. Men were more often married or living with a partner, had a high level of education and reported a higher score on the life satisfaction Likert scale. Moreover, men more often seem to have made experience with a serious illness themselves, while women reported more experience with illness in their families.

**Table 1:** Study sample characteristics as compared to the German elderly reference population

Sample	Total N = 290	Men N = 132	Women N = 158	Elderly German reference population*	
				Men 42.5%	Women 57.5%
Age, mean (SD)	73.1 (5.7)	72.5 (5.4)	73.6 (5.8)		
Range	65–93	65–93	65–90		
Age groups in years, N (%)					
65–69 70–74 75–79 80 +	86 (29.7) 78 (26.9) 88 (30.3) 38 (13.1)	43 (32.6) 37 (28.0) 43 (32.6) 9 (6.8)	43 (27.2) 41 (26.0) 45 (28.5) 29 (18.3)		
				25.7% 29.0% 45.3%	21.4% 25.4% 53.2%
				(Last two groups combined)	
Marital status, N (%)					
Married/living with a partner Single Divorced/separated Widowed	157 (54.1) 14 (4.8) 40 (13.8) 79 (27.2)	95 (72.0) 7 (5.3) 13 (9.9) 17 (12.9)	62 (39.2) 7 (4.4) 27 (17.1) 62 (39.2)	73.3% 5.4% 9.4% 11.7%	47.9% 4.2% 10.6% 37.4%
Educational attainment, N (%)					
Low Middle High	179 (61.7) 56 (19.3) 55 (19.0)	72 (54.6) 22 (16.7) 38 (28.8)	107 (67.7) 34 (21.5) 17 (10.8)	54.3% 19.9% 25.4%	62.2% 25.0% 12.5%
Region, N (%)					
Berlin Leipzig Hamburg Bielefeld Munich Frankfurt	35 (12.1) 76 (26.2) 47 (16.2) 45 (15.5) 56 (19.3) 31 (10.7)	13 (9.9) 35 (26.5) 23 (17.4) 19 (14.4) 30 (22.7) 12 (9.1)	22 (13.9) 41 (26.0) 24 (15.2) 26 (16.5) 26 (16.5) 19 (12.0)		
Area of residence, N (%)					
Urban Rural	198 (68.3) 92 (31.7)	93 (66.4) 39 (29.6)	105 (66.5) 53 (33.5)	65.4% 34.6%	
Life satisfaction, N (%)					
0–3 4–6 7–10	4 (1.4) 36 (12.4) 250 (86.2)	1 (0.8) 10 (7.6) 121 (91.7)	3 (1.9) 26 (16.5) 129 (81.7)		
Experience with serious illness, N (%)					
Yes No	136 (47.0) 154 (53.0)	71 (53.8) 61 (46.2)	65 (41.1) 93 (58.9)		
Experience with serious illness in family, N (%)					
Yes No	185 (63.8) 105 (36.2)	76 (57.6) 56 (42.4)	109 (69.0) 49 (31.0)		

References: [33, 47–49]

### EQ-5D-5L dimensions

In total, 93 unique health states were reported with the three most frequent being ‘11111’ (21.4%), ‘11121’ (14.5%) and ‘21121’ (6.2%). No respondent reported to be in the pits state ‘55555’. Table 2 presents the frequency of reported problems for each dimension by age groups for the total sample, whereas Tables 3 and 4 present the results for men and women respectively. The distribution of reported problems is uneven across dimensions. Problems were most frequently reported for pain or discomfort with 68.3% of the total sample reporting any problems, while problems with self-care were the least frequent with only 15.5% of respondents reporting any problems. Problems with mobility, usual activities and anxiety or depression were reported by 52.1%, 35.2% and 27.6% respectively. Overall, extreme problems were rarely reported; the share of extreme problems/ unable to is less than 2% in any dimension (Table 2).

**Table 2:** Reported problems in EQ-5D-5L by age groups for the total sample

Parameter	Age								Total	
	65–69		70–74		75–79		80 +			
	n	%	n	%	n	%	n	%	n	%
Total										
N	86		78		88		38		290	
**Mobility**										
No problems	58	67.4	39	50.0	32	36.4	10	26.3	139	47.9
Slight problems	17	19.8	18	23.1	22	25.0	6	15.8	63	21.7
Moderate problems	7	8.1	14	18.0	19	21.6	10	26.3	50	17.2
Severe problems	4	4.7	7	9.0	15	17.1	12	31.6	38	13.1
Unable to	0	0.0	0	0.0	0	0.0	0	0.0	0	0
**Self-care**										
No problems	82	95.4	66	84.6	74	84.1	23	60.5	245	84.5
Slight problems	2	2.3	9	11.5	8	9.1	4	10.5	23	7.9
Moderate problems	2	2.3	2	2.6	4	4.6	5	13.2	13	4.5
Severe problems	0	0.0	1	1.3	2	2.3	4	10.5	7	2.4
Unable to	0	0.0	0	0.0	0	0.0	2	5.3	2	0.7
**Usual activities**										
No problems	66	76.7	54	69.2	52	59.1	16	42.1	188	64.8
Slight problems	15	17.4	9	11.5	19	21.6	7	18.4	50	17.2
Moderate problems	3	3.5	12	15.4	14	15.9	5	13.2	34	11.7
Severe problems	2	2.3	2	2.6	2	2.3	7	18.4	13	4.5
Unable to	0	0.0	1	1.3	1	1.1	3	7.9	5	1.7
**Pain/discomfort**										
No	39	45.4	20	25.6	23	26.1	10	26.3	92	31.7
Slight	35	40.7	31	39.7	33	37.5	13	34.2	112	38.6
Moderate	10	11.6	20	25.6	23	26.1	8	21.1	61	21.0
Severe	1	1.2	7	9.0	9	10.2	6	15.8	23	7.9
Extreme	1	1.2	0	0.0	0	0.0	1	2.6	2	0.7
**Anxiety/depression**										
No	68	79.1	60	76.9	63	71.6	19	50.0	210	72.4
Slight	16	18.6	11	14.1	17	19.3	11	29.0	55	19.0
Moderate	2	2.3	7	9.0	8	9.1	5	13.2	22	7.6
Severe	0	0.0	0	0.0	0	0.0	2	5.3	2	0.7
Extreme	0	0.0	0	0.0	0	0.0	1	2.6	1	0.3

**Table 3:** Reported problems in EQ-5D-5L by age groups for male respondents

Parameter	Age								Total	
	65–69		70–74		75–79		80 +			
	n	%	n	%	n	%	n	%	**n**	**%**
Total										
N	43		37		43		9		132	
**Mobility**										
No problems	28	65.1	21	56.8	17	39.5	3	33.3	69	52.3
Slight problems	10	23.3	6	16.2	9	20.9	1	11.1	26	19.7
Moderate problems	3	7.0	7	18.9	9	20.9	3	33.3	22	16.7
Severe problems	2	4.7	3	8.1	8	18.6	2	22.2	15	11.4
Unable to	0	0.0	0	0.0	0	0.0	0	0.0	0	0.0
**Self-care**										
No problems	41	95.4	31	83.8	35	81.4	5	55.6	112	84.9
Slight problems	1	2.3	5	13.5	4	9.3	2	22.2	12	9.1
Moderate problems	1	2.3	0	0.0	2	4.7	2	22.2	5	3.8
Severe problems	0	0.0	1	2.7	2	4.7	0	0.0	3	2.3
Unable to	0	0.0	0	0.0	0	0.0	0	0.0	0	0.0
**Usual activities**										
No problems	34	79.1	27	73.0	25	58.1	5	55.6	91	68.9
Slight problems	6	14.0	2	5.4	9	21.0	2	22.2	19	14.4
Moderate problems	2	4.7	6	16.2	7	16.3	1	11.1	16	12.1
Severe problems	1	2.3	1	2.7	1	2.3	1	11.1	4	3.0
Unable to	0	0.0	1	2.7	1	2.3	0	0.0	2	1.5
**Pain/discomfort**										
No	22	51.2	12	32.4	10	23.3	2	22.2	46	34.9
Slight	18	41.9	12	32.4	17	39.5	6	66.7	53	40.2
Moderate	3	7.0	9	24.3	11	25.6	1	11.1	24	18.2
Severe	0	0.0	4	10.8	5	11.6	0	0.0	9	6.8
Extreme	0	0.0	0	0.0	0	0.0	0	0.0	0	0.0
**Anxiety/depression**										
No	36	83.7	31	83.8	36	83.7	7	77.8	110	83.3
Slight	6	14.0	5	13.5	5	11.6	2	22.2	18	13.6
Moderate	1	2.3	1	2.7	2	4.7	0	0.0	4	3.0
Severe	0	0.0	0	0.0	0	0.0	0	0.0	0	0.0
Extreme	0	0.0	0	0.0	0	0.0	0	0.0	0	0.0

**Table 4:** Reported problems in EQ-5D-5L by age groups for female respondents

Parameter	Age								Total	
	65–69		70–74		75–79		80 +			
	n	%	n	%	n	%	n	%	n	%
Total										
N	43		41		45		29		158	
**Mobility**										
No problems	30	69.8	18	43.9	15	33.3	7	24.1	70	44.3
Slight problems	7	16.3	12	29.3	13	28.9	5	17.2	37	23.4
Moderate problems	4	9.3	7	17.1	10	22.2	7	24.1	28	17.7
Severe problems	2	4.7	4	9.8	7	15.6	10	34.5	23	14.6
Unable to	0	0.0	0	0.0	0	0.0	0	0.0	0	0.0
**Self-care**										
No problems	41	95.4	35	85.4	39	86.7	18	62.1	133	84.2
Slight problems	1	2.3	4	9.8	4	8.9	2	6.9	11	7.0
Moderate problems	1	2.3	2	4.9	2	4.4	3	10.3	8	5.1
Severe problems	0	0.0	0	0.0	0	0.0	4	13.8	4	2.5
Unable to	0	0.0	0	0.0	0	0.0	2	6.9	2	1.3
**Usual activities**										
No problems	32	74.4	27	65.9	27	60.0	11	37.9	97	61.4
Slight problems	9	20.9	7	17.1	10	22.2	5	17.2	31	19.6
Moderate problems	1	2.3	6	14.6	7	15.6	4	13.8	18	11.4
Severe problems	1	2.3	1	2.4	1	2.2	6	20.7	9	5.7
Unable to	0	0.0	0	0.0	0	0.0	3	10.3	3	1.9
**Pain/discomfort**										
No	17	39.5	8	19.5	13	28.9	8	27.6	46	29.1
Slight	17	39.5	19	46.3	16	35.6	7	24.1	59	37.3
Moderate	7	16.3	11	26.8	12	26.7	7	24.1	37	23.4
Severe	1	2.3	3	7.3	4	8.9	6	20.7	14	8.9
Extreme	1	2.3	0	0.0	0	0.0	1	3.5	2	1.3
**Anxiety/depression**										
No	32	74.4	29	70.7	27	60.0	12	41.4	100	63.3
Slight	10	23.3	6	14.6	12	26.7	9	31.0	37	23.4
Moderate	1	2.3	6	14.6	6	13.3	5	17.2	18	11.4
Severe	0	0.0	0	0.0	0	0.0	2	6.9	2	1.3
Extreme	0	0.0	0	0.0	0	0.0	1	3.5	1	0.6

Visual inspection showed that women tended to report more problems with mobility, usual activities and pain or discomfort than men (Tables 3 and 4). Problems with anxiety or depression were the second least prevalent in the overall sample. But then again, women reported significantly more problems with anxiety or depression when compared to men (36.7% vs. 16.7%).

The prevalence of reported health problems on EQ-5D-5L increases almost monotonically with age reaching the highest share in the age group 80 + years, indicating that the EQ-5D dimensions seem to be sensitive towards age-related health problems. Pain or discomfort is the exception to this pattern, starting with a high level of reported problems, but remained at a similar level beyond the age of 70 years.

### EQ-5D-5L index population norms

**Fig. 1:**
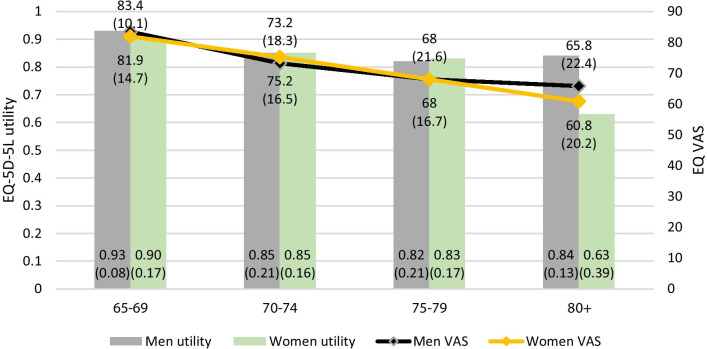
German EQ-5D-5L average utility and VAS values by age groups and gender

Table 5 provides the EQ-5D-5L index values for the total sample further stratified by gender and age groups, presented as means with standard deviations and 95% confidence intervals. The mean index score for the total sample was 0.84 (SD 0.012, 0.814–0.864 95% CI). The index values ranged from − 0.485 to 1. While only three respondents had negative utilities, 54% of the total sample (n = 157) had a utility value of ≥ 0.9. EQ-5D-5L index values were non-normally distributed (p < 0.01). On average, men had a higher index score than women (mean 0.87 (SD 0.18) vs. 0.82 (0.24))—the difference was statistically significant at the 5% level (p = 0.027). Further, mean utilities consistently decrease with increasing age; the mean utility for the age group 65–69 years was 0.92, while it decreased to 0.68 for the oldest age group (see Table 5). The mean index values across age groups differed significantly (p < 0.01). For both men and women the mean utility is highest in the youngest age group (65–69 years) and slightly decreased after the age of 69 years, again being at a similar level for both. However, while the mean index appears stable for men with progressing age, women reported a significant deterioration in mean utility in the oldest age group (p < 0.05) (see Fig. 1).

**Table 5:** EQ-5D-5L index population norms by age groups and gender based on the German EQ-5D-5L tariff by Ludwig et al. (2018)

EQ-5D-5L index value	Age				Total
	65–69	70–74	75–79	80 +	
**Total**					
N	86	78	88	38	290
Mean	0.92	0.85	0.82	0.68	0.84
Standard deviation	0.13	0.19	0.19	0.35	0.22
95% CI–lower bound	0.888	0.807	0.785	0.566	0.814
95% CI–upper bound	0.945	0.890	0.864	0.793	0.864
**Men**					
N	43	37	43	9	132
Mean	0.93	0.85	0.82	0.84	0.87
Standard deviation	0.08	0.21	0.21	0.13	0.18
95% CI–lower bound	0.910	0.781	0.752	0.757	0.835
95% CI–upper bound	0.958	0.921	0.881	0.931	0.898
**Women**					
N	43	41	45	29	158
Mean	0.90	0.85	0.83	0.63	0.82
Standard deviation	0.17	0.16	0.17	0.39	0.24
95% CI–lower bound	0.847	0.796	0.783	0.486	0.779
95% CI–upper bound	0.950	0.896	0.881	0.770	0.854

### EQ-VAS population norms

Table 6 presents reference values based on the EQ VAS for the total sample and stratified for gender and age groups. Norms data are presented as means, standard deviation and 95% confidence interval around the mean. Again, the EQ VAS values were non-normally distributed. Overall, self-reported EQ VAS values ranged from 10 to 100 with the three most frequently reported values being 90 (17.9%), 80 (14.8%) and 50 (12.8%). The mean reported EQ VAS for the total sample was 73.2 (SD 18.5, 71.1–75.4 95% CI). When compared to women, men reported an average EQ VAS value that was two points higher (mean 74.3 (SD 18.7) vs. 72.3 (18.3)). However, this difference was not statistically significant (p = 0.27).

**Table 6:** EQ VAS population norms by age groups and gender

EQ-5D-5L VAS value	Age				Total
	65–69	70–74	75–79	80 +	
**Total**					
N	86	78	88	38	290
Mean	82.6	74.3	68.0	61.9	73.2
Standard deviation	12.6	17.3	19.1	20.5	18.5
95% CI–lower bound	80.0	70.4	64.0	55.4	71.1
95% CI–upper bound	85.3	78.1	72.0	68.5	75.4
**Men**					
N	43	37	43	9	132
Mean	83.4	73.2	68.0	65.8	74.3
Standard deviation	10.1	18.3	21.6	22.4	18.7
95% CI–lower bound	80.3	67.2	61.4	51.0	71.1
95% CI–upper bound	86.4	79.2	74.5	80.5	77.5
**Women**					
N	43	41	45	29	158
Mean	81.9	75.2	68.0	60.8	72.3
Standard deviation	14.7	16.5	16.7	20.2	18.3
95% CI–lower bound	77.5	70.1	65.1	53.3	69.5
95% CI–upper bound	86.3	80.3	73.0	68.2	75.2

Similar to the computed utility reference values, mean EQ VAS monotonically decreased with increasing age starting with 82.6 (SD 12.6) for the age group 65–69 years further declining to 61.9 (SD 20.5) in the oldest age group. Again, mean EQ VAS differed across age groups (p < 0.01). Considering age and gender jointly, EQ VAS reference values followed a similar trend as the utility norm values. However, the difference between 80-year-old men and women was less pronounced (see Fig. 1).

## Discussion

The aim of this study was to provide population norms for the German population aged 65 years and above. To the best of our knowledge this is the first study to provide reference values for the elderly population in Germany, which is based on the recently developed tariff for the German version of the EQ-5D-5L [32].

The overall mean utility score in our study was slightly lower than the reported overall value for the German general population (0.84 vs. 0.88), which included all age groups [19]. However, the mean index reported here is considerably higher than that of the oldest-old in Germany as reported by König et al. [23] (0.84 vs. 0.77(GER)/ 0.68(UK)). This finding replicates the negative association of age with mean utility scores, which was found in other population norm studies as well [14, 21, 27, 36–38]. Another source for the deviation may be the choice of the instrument and value set.

Conventional population health studies only use two age categories to represent the elderly population, which we further split into two smaller age bands to facilitate a more detailed comparison based on age groups. When comparing the computed mean utilities with the values reported for the German elderly by Grochtdreis et al. [19], we find that the two middle categories (70–74 and 75–79 years) largely agree with the broader values reported for Germany. On the contrary, mean index values of those in the age group 65–69 years are considerably higher, whereas the opposite is the case for the oldest old [19]. This may indicate that the decline in HRQoL that is associated with high age is even more pronounced than may be assumed based on the broader age categories from conventional population norm studies. Evidence of a considerable decline beyond the age of 80 can be found in other studies, as well [23, 27, 29, 39].

Furthermore, men reported similar or higher mean utility than women. This difference was largest in the oldest age category. Again, this pattern can be confirmed for Germany [19, 23, 38]. However, Hinz et al. [38] calculated sum scores based on the severity levels of the descriptive system rather than utilities, which limits the comparability. The observed negative association of increasing age and female sex with HRQoL was also found when comparing mean EQ VAS values, which was also found internationally [17]. On average, men reported higher EQ VAS values than women (74.2 vs. 72.3) and this was consistent across age groups. The only exception were women aged 70–74 years reporting higher EQ VAS values than their male counterparts. Similar findings were described by Huber et al. [28] for Germany.

Considering the health profile level, 21.4% of this elderly sample reported to have no problems in all dimensions, which is considerably less than the proportions found in other German studies for the general population [19, 28, 38] and comparable to the share reported by König et al. [23] for the German oldest-old. Generally, the observed response distribution in this study largely adheres to that observed for similar age categories in another study for Germany [19] and internationally [14, 21, 27, 36]. Overall, we observed an increasing prevalence of reported health problems with higher age. In this sample, problems were most frequently reported in the dimensions of pain or discomfort and mobility, whereas only 15.5% of the total sample reported problems with self-care. Such a pattern, where age-related health impairments seem to predominantly manifest as problems with pain or discomfort and mobility, was also observed elsewhere [19, 23, 29, 40]. The diminished proportion of respondents at the ceiling in our sample of the elderly seems to indicate that the EQ-5D-5L is sensitive towards age-related health problems. This aligns well with the argument made by Konnopka and König [41] that ceiling effects decrease with higher levels of morbidity, which are regularly observed in samples of the elderly [19, 25, 27, 29, 36, 42].

There is a wide body of population norms indicating that problems with anxiety or depression remain at a similar level independent of age [14, 19, 21, 25, 29, 36, 40, 43]. However, this was only the case for men in our sample; women, on the other hand, reported more problems with an increasing rate at older age. Overall, the rate of reported problems with anxiety and depression increased with increasing age similar to the other dimensions, but we found this trend to be driven by the higher proportion of women in the higher age groups who tend to report more problems with anxiety or depression [23, 27, 29]. Interestingly, Jiang et al. [18] and others found evidence of a decreasing prevalence of problems with anxiety or depression in older respondents [20, 44], which they explained with social stigma being attached to mental health problems and, hence, may lead to a lower frequency of acknowledged mental health problems. A similar response pattern was also described by König et al. [23] for the oldest-old (85 +) using the EQ-5D-3L.

One strength of this study is the use of data from a sample of German general population, which is representative in terms of age, gender, education, employment status and area of residence. Further, this study provides reference data on all three information components of the EQ-5D-5L. Importantly, the index values were derived using the recommended tariff by Ludwig et al. [32] providing additional information compared to earlier studies, which described health of the German population based on the EQ-5D-5L, but using unweighted sum scores [38] or EQ VAS values [28]. Furthermore, we provide references values for smaller age bands, which may enable a more detailed comparison when using these references. However, some limitations of this study must be considered. While information on respondents’ experience with severe illness is available, the data lacks detailed information on prevalent long-term conditions or comorbidities. Since the data was collected as part of the German EQ-5D-5L valuation study, where respondents engage in a cognitively demanding task, it can be assumed that this sample may be cognitively and physically healthier than older people who are not participating in valuation interviews. Similarly, due to the primary purpose of the underlying data set, respondents were not sampled to represent the German elderly population per se, but to represent the general population; by this, individuals living in institutions, such as residential aged care facilities, may be underrepresented. Both of these limitations may have introduced a selection bias, which potentially led to an overestimation of the elderly population’s health by this sub-sample. Moreover, the sample size can be considered small for an EQ-5D-5L reference value study. Therefore, the precision in the confidence intervals for the means is relatively low, leading to some overlap in CIs between adjacent age groups, which limits the certainty of detecting true differences between age groups in mean EQ VAS and EQ-5D-5L index values. Secondly, reference values for the oldest age group are based on very few observations (i.e. n = 38). Thus, the robustness and generalisability of the reference value for this age group are limited and should be used cautiously.

Due to the secondary nature of this data set, the sample size results as a consequence to the EQ-5D-5L valuation protocol with a target of N = 1000 respondents [45, 46], where this sub-sample represents the proportionate share of the elderly population from the original data. While we believe this to be an efficient use of existing data to generate benchmark values, future research may take the special characteristics of the older population into account and improve in sample size to increase generalisability and precision of the results.

## Conclusion

These values were derived from a representative sample of the German elderly using the recommended tariff for the EQ-5D-5L. The findings may enable empirical comparisons of EQ-5D-5L based HRQoL with other samples, to assess change in health over time or burden of disease. Generally, the observed mean utilities and VAS values as well as the dimension-level response distribution correspond well to earlier findings from a large German population norm study. However, a more detailed provision of reference values for the elderly population seems helpful given that HRQoL in the oldest old is less consistent and falls off in comparison to the young elderly, while the sought age categorisation of conventional population norms studies seems to mask these differences. Hence, our findings may facilitate a more precise comparison across elderly age groups. Nevertheless, we suggest conducting further research to explore aspects and determinants of HRQoL for the age group above 80 years.

## Data Availability

The datasets generated during and/or analyzed during the current study are available from the corresponding author on reasonable request.

## Abbreviations

EQ VAS: EQ-5D visual analogue scale
SD: Standard deviation
HRQoL: Health-related quality of life
CI: Confidence interval
MO: Mobility
SC: Self-care
UA: Usual activities
PD: Pain or discomfort
AD: Anxiety or depression
